# Economic evaluation of financial incentives for maternal and child health in the Democratic Republic of the Congo (DRC): a decision-tree modelling based on a cluster randomized controlled trial

**DOI:** 10.1186/s41256-025-00435-9

**Published:** 2025-09-01

**Authors:** Wu Zeng, Gil Shapira, Tianjiao Gao, Michel Muvudi, Guohong Li, Jennifer Bouey, Delphin Antwisi, Fatma El Kadiri El Yamani

**Affiliations:** 1 Department of Global Health, Georgetown University, Washington, DC USA; 2 World Bank, Washington, DC USA; 3 World Bank Country Office in the Democratic Republic of the Congo, Kinshasa, Democratic Republic of the Congo; 4 School of Global Health, Shanghai Jiaotong University School of Medicine, Shanghai, China; 5 Project Implementation Unit of World Bank Health Project, Ministry of Public Health, Hygiene and Prevention, Kinshasa, Democratic Republic of the Congo

**Keywords:** Performance-based financing, Pay for performance, Cost-effectiveness

## Abstract

**Background:**

To improve the utilization of maternal and child health (MCH) services, the Democratic Republic of the Congo initiated a performance-based financing (PBF) program in 2017. An impact evaluation of the PBF program was conducted in 2023 using a cluster randomized controlled trial research design. This study aimed to assess the cost-effectiveness of the PBF program in comparison with the direct facility financing (DFF) program.

**Methods:**

A decision-tree model incorporating key MCH services was developed to estimate cost-effectiveness. Data on costs of maternal health services, epidemiological consequences, and utilities of various health statuses were obtained from the literature. The impact evaluation results on the coverage of key MCH services were included as key inputs to simulate the effectiveness measured as quality-adjusted life years (QALYs). Sensitivity analyses were conducted on the inclusion of the PBF’s impact on the quality of care and the uncertainty regarding the costs and the impact of PBF on MCH services.

**Results:**

A total of US$205.9 million in 2021 dollar was spent on the PBF arm over the five years (2017–2021), with 70.60% allocated as incentive payments to health facilities and 19.41% as financial transfers to provincial purchasing agencies for contracting PBF facilities and managing the PBF program. On average, the annual cost per capita was estimated at US$2.05 and US$1.71 for implementing the PBF and DFF program, respectively. Without the quality adjustment, the improvement in MCH services resulted in 1,372 lives (192,036 QALYs) saved over 2017–2021. The incremental cost-effectiveness ratio (ICER) of the PBF program reached US$ 1,374 per QALY with substantial variation. After adjusting for quality, the ICER of PBF became smaller.

**Conclusions:**

Using three times the gross domestic product per capita in 2021 (US$1,732) as the threshold, the PBF program is a cost-effective strategy, though with substantial variation. It is crucial to take action to maintain gains from the improved MCH coverage resulting from the PBF program.

**Supplementary Information:**

The online version contains supplementary material available at 10.1186/s41256-025-00435-9.

## Introduction

Despite continuous economic growth in the Democratic Republic of the Congo (DRC) in recent years, the DRC consistently ranks among countries with the worst maternal and child health (MCH) indicators globally, with an under-5 mortality rate of 79.0 per 1,000 births in 2021 and a maternal mortality rate of 547 per 100,000 live births in 2020 [1]. Poor quality of care, inadequate availability of essential MCH services, lack of access to emergency services, and the absence of an effective referral system to a higher level of care are among the main causes of the high under-5 and maternal mortality rates [2–4].

Meanwhile, the central government contributed only 18% of the total health expenditure, [5] and DRC has struggled to mobilize additional resources for the health sector. Most health facilities fail to receive financing and resources on a routine basis [6]. The country lacks the funds to provide salaries and relies heavily on charging high user fees to compensate staff and pay for supplies and maintenance costs. The low morale of the health workforce exacerbates the low quality of care in the country [7–10].

To address some of the above-mentioned health system’s bottlenecks, the DRC government implemented a performance-based financing (PBF) program in 12 provinces covering more than 100 health zones in 2016, as part of the Health System Strengthening for Better Maternal and Child Health Result Project (*PDSS – Le Projet de Développement du Système de Santé*). PBF provides financial incentives to administrative units and/or health facilities based on predefined indicators (e.g., the quantity and quality of health services). It has been implemented in many countries as a way to use scarce resources more efficiently, provide care for hard-to-reach groups, and encourage providers to reduce user fees [11–15].

Along with the PBF program, DRC implemented a direct facility financing (DFF) program alongside as control. DFF, which provided health facilities with direct financial resources without conditionalities, has also gained increasing popularity [16]. In Zambia and Nigeria, PBF was found to be more effective in improving health services than DFF for some services, but at a higher cost, partially due to activities such as verification and counter-verification [17, 18]. While there has been an increasing number of studies examining the impact of the PBF program, most studies compared the PBF program to the business-as-usual model with mixed results [19]. The cost-effectiveness of the PBF programs was even more limited. A total of six studies concerning the cost-effectiveness of PBF programs were found [18, 20–24], and only three of them compared PBF with DFF programs [18, 22, 23]. This economic evidence is critical. For key policymakers, an important policy question arises: is it worthwhile designing and implementing a more costly PBF program as opposed to leveraging the country’s existing system to implement a DFF program?

An impact evaluation of the PBF program in DRC, in comparison with DFF, was conducted using both household and health facilities surveys, which showed the potential benefits of using PBF to improve both the quality and coverage of MCH services [25]. Building on this impact evaluation, this study uses expenditure and cost data from the program implementation database and literature to evaluate the cost-effectiveness of the PBF program compared with DFF. This study aims to contribute economic evidence to the limited existing literature on the economic evaluation of PBF programs.

## Methods

### Study design

This cost-effectiveness analysis (CEA) leveraged the results from the impact evaluation of the PBF program conducted between 2017 and 2021 [25]. The impact evaluation used a cluster-randomized controlled trial design at the health zone level, randomizing a total of 100 health zones across 11 provinces (excluding one province in the pre-pilot phase) into either the PBF group or the control group. All health facilities in the selected PBF health zones received quarterly incentive payments based on their performance, in addition to the budget allocated by the government. The incentivized indicators focused on maternal and child health (MCH) services, including child vaccination, antenatal care (ANC), institutional delivery (ID), postnatal care (PNC), family planning, growth monitoring, as well as HIV and tuberculosis services, minor surgeries, referrals, and outpatient visits. The detailed indicators and their fee schedules are described in the impact evaluation study [25, 26]. Up to 50% of the incentive payments could be allocated to health staff as bonuses, while a minimum of 20% was required to be spent on medications and other consumables to improve service delivery. Health facilities in the control health zones (referred to as the DFF arm/zone) received unconditional quarterly payment transfers equivalent to the average payments made to experimental facilities (PBF arm) in the same zone, adjusted for catchment area population size. A more detailed description of the PBF program and its study design has been published elsewhere [25, 26].

### Perspective of the cost-effectiveness analysis

The reporting of this CEA follows the Consolidated Health Economic Evaluation Reporting Standards 2022 (CHEERS 2022) [27]. The CEA was conducted from a health system perspective for the PBF program between 2017 and 2021. As the CEA was conducted from the health system perspective rather than societal perspective, and only captured costs from health providers, the direct non-medical costs of seeking care incurred by households (e.g., transport and accommodation) and indirect costs due to patient productivity loss borne by society were not included in the analysis [18, 28].

### Decision tree for the cost-effectiveness analysis

**Fig. 1:**
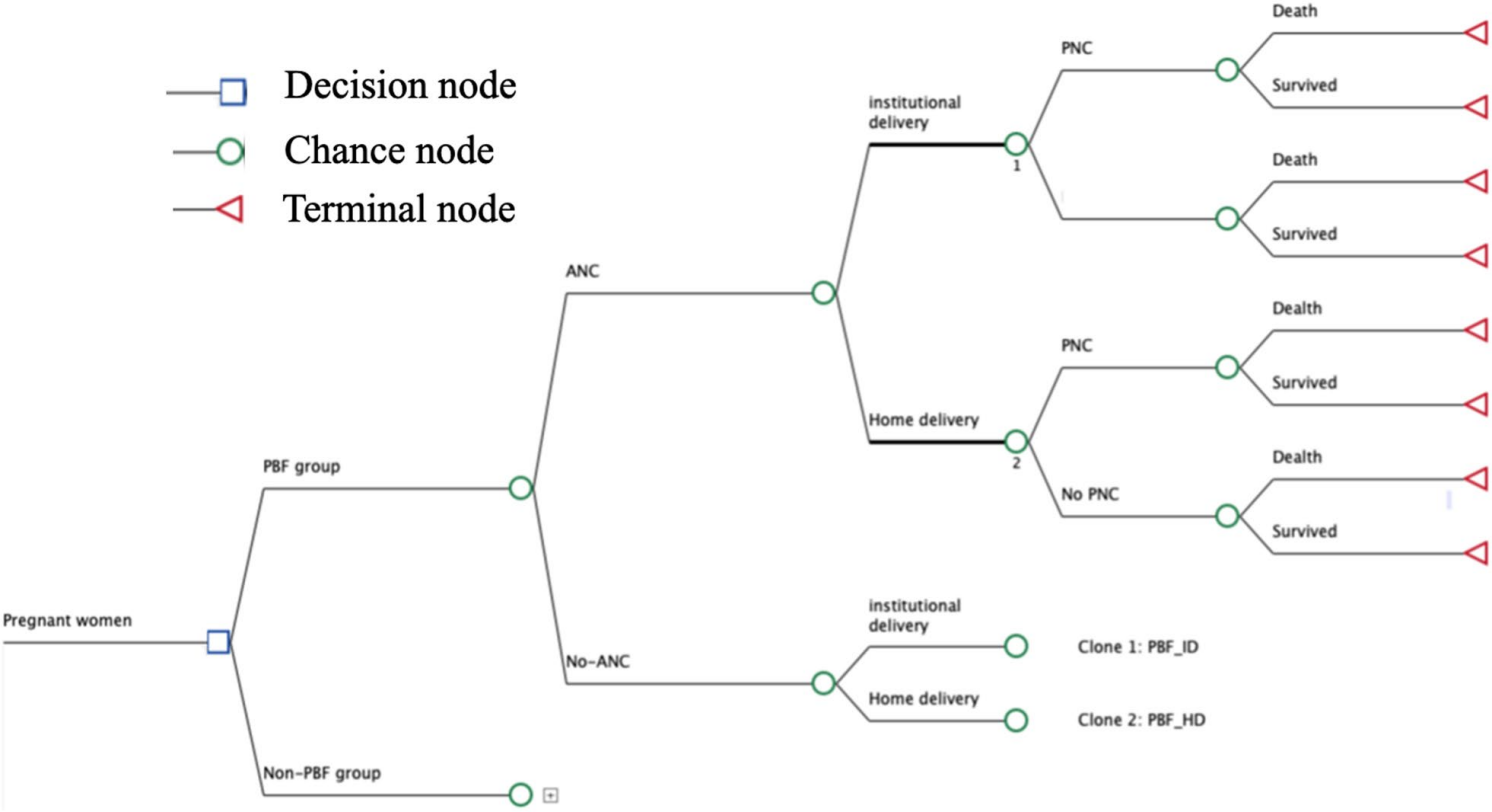
Decision tree for the cost-effectiveness analysis

Based on the impact evaluation results from a cluster randomized research design [25] and the prior frameworks to assess the cost-effectiveness of MCH interventions [29], we developed a decision tree for maternal and newborn care. The decision tree is presented in Fig. 1.

The decision tree used the following three major indicators: 4 + times ANC visits, ID, and PNC visits. Given that the PBF did not have any impact on the coverage of vaccines among children under 2 years old, vaccines were excluded from the analysis [25]. A more detailed justification for the selection indicators for the model is provided in the effectiveness assessment section.

### Cost assessment

In this analysis, we focused on the incremental cost incurred in the PBF versus DFF arms. To capture costs in the two groups, we included the following in the analysis: (I) program implementation costs, (II) costs of delivering the three key maternal health services (e.g., ANC, ID, and PNC), and (III) costs for providing technical assistance to both PBF and direct financing health zones, including program design, implementation, and monitoring. Costs were collected or estimated for the period between January 2017 and December 2021 for both groups. The cost difference between the two groups was then calculated as the incremental cost for the CEA.

Specifically, program implementation costs for the two groups were obtained from project disbursement data compiled by the World Bank. The data include information on funding disbursements to health facilities (incentive payments to PBF facilities or unconditional payments to DFF facilities), health zone teams, and provincial health teams for general management in both the PBF and DFF arms per year. Cost items specific to the PBF arm were also captured, such as payments to provincial health teams for performance evaluation at hospitals, counter-verification, and support for purchasing services from PBF facilities (strategic purchasing), as well as financial disbursements to the provincial purchasing agency to contract with PBF facilities and manage the PBF program. However, one expenditure item was missing in the DFF arm (financial transfers to health zone teams in 2018), and we replaced it with the average spending in 2017 and 2019 after consulting with program implementers. We also replaced the disbursement to DFF health facilities for the last quarter of 2019 with the average of disbursements from the first three quarters of 2019, as the disbursement in the last quarter was abnormally high.

For the costs of providing ANC, ID, and PNC, we obtained unit costs for these services from the literature (Table 2). For cost data (e.g., unit costs for ANC and PNC) from countries other than the DRC, costs were adjusted for gross domestic product (GDP) per capita in the year the cost was estimated, using a log-linear regression approach with an established relationship between GDP per capita and the respective unit cost [30]. In addition to the unit costs for the three services, we also obtained the cost of treating birth delivery complications from the literature for the DRC.

No cost information was available specifically for technical assistance to the PBF and DFF arms for project planning and management. Data on technical support were available for the overall PDSS project, which includes the PBF program and other interventions, totaling US$12.5 million. We used the share of program expenditure for the PBF project (including expenditures for the PBF and DFF arms) as a percentage of the total PDSS budget to estimate the cost of technical assistance for the PBF project (25.68%). To allocate the estimated cost for technical assistance between the PBF and DFF arms, we used the ratio of program implementation costs for the PBF arm to those for the DFF arm. All the costs were converted to 2021 US$ after adjusting for inflation. Using the population size in each arm, the cost of program implementation and technical support was estimated as the cost per capita per year in each arm, divided by the population size in the specific year, and was used in the decision tree model.

### Effectiveness assessment

The World Bank conducted an impact evaluation of nine indicators on service coverage using surveys from both households and health facilities through a clustered randomized controlled trial [25]. These nine indicators include four on ANC, one on ID, one on PNC, one on family planning, one on growth monitoring, and one on vaccination. The evaluation shows that PBF has the potential to improve maternal and child health services.

In reviewing the evaluation result, we selected the following indicators for the modelling: 4 + times ANC visits, ID, and any PNC. These three indicators were the key incentivized maternal health services under the PBF, and the selection is consistent with the decision tree in Appendix 1. While there were four indicators of ANC in the impact evaluation, we selected 4 + times ANC in the CEA because it was reported that 4 + times ANC has the most important contribution to maternal and child health [31].

We excluded vaccination, family planning, and growth monitoring indicators from the study for three reasons: (1) there was no difference in vaccination coverage between DFF and PBF arms, (2) growth monitoring lacked a clear pathway for affecting maternal and child health outcomes, and (3) family planning data lacked specificity regarding contraceptive types. The study also demonstrated that PBF might improve quality of care for these three maternal health services. We included PBF's quality-of-care impact in our sensitivity analysis. Table 1 presents PBF's impact on both coverage and quality for these three services.

**Table 1:** Baseline and impact of PBF on the coverage and quality of care for antenatal care (ANC), institutional delivery (ID), and postnatal care (PNC). Source: Shapira et al. 2023

Indicator	Mean in DFF group	Impact	% Change
Coverage			
4 + ANC	0.34	0.03	9.0%
ID	0.91	0.03	3.0%
Any PNC	0.39	0.03	8.0%
Quality of care (process quality)			
ANC score	0.91	0.02	3.0%
ID score	0.60	0.03	5.0%
PNC score	0.07	0.03	45.0%

The impact assessment presented baseline coverage of health services and the PBF program's impact on coverage. To simplify the modeling, we adjusted the baseline by equalizing coverage between the PBF and DFF arms, then recalculated ANC, ID, and PNC coverage by year, assuming linear increases/decreases over the five-year period. We used the midpoint coverage as the annual coverage estimate across all five years to avoid impact overestimation.

### Cost-effectiveness modelling

To account for differences in population size, baseline coverage, and quality of health services between the PBF and DFF programs, we used the PBF arm's population size (18.5, 19.0, 19.9, 20.0, and 23.0 million from 2017 to 2021, respectively) as the standardized population size for the CEA. We also used the DFF arm's baseline coverage of MCH services as the standardized baseline coverage for both arms.

To apply the decision model using the overall coverage of ANC, ID, and PNC, a key step was deriving the conditional probability for each branch and the conditional death rate for the terminal nodes in the decision tree. We obtained odds ratios and treated them as a risk ratio for utilizing specific health services given a prior health service was provided (e.g., odds ratio of using ID for those who had more than 4 times ANC visits). Using a Bayesian approach, we derived conditional probabilities for each branch. Similarly, we estimated conditional maternal and neonatal mortality rates annually by using the national maternal and neonatal death rate and the odds ratios of deaths for those who received maternal services vs those who did not.

For each year and study arm, we estimated maternal and neonatal deaths using the decision tree model, standardizing the population size to that of the PBF arm. We then calculated lives saved among pregnant women and newborns over the five-year period. We obtained the life expectancy for pregnant women at death and at birth to estimate the quality-adjusted life years (QALYs) lost per death using the following formula [18, 32]:

$$\text{QALYs loss per death}= Q*\frac{1-{e}^{-r\left({L}_{a}\right)}}{r}$$

where Q is the average quality of life if one survives, *r* is the annual discount rate, which was commonly set at 3% [33], and *L*_*a*_ is life expectancy at the age of death of *a*. It was estimated that 21.88 QALYs were lost per pregnant woman death and 24.34 QALYs per newborn death in 2021, respectively. There is no data on the average quality of life in DRC. We estimated Q as the ratio of healthy-adjusted life expectancy to life expectancy at birth in DRC, which was 0.86 [34]. The total QALY gained due to the PBF program was the sum of QALY gains for pregnant women and that for newborns.

An ICER was estimated using the difference in cost and the QALYs gained between the PBF and DFF arms. Table 2 details all parameters and data sources for the CEA.

**Table 2:** Key parameters for the cost-effectiveness modelling

Parameters	Value	Standard deviation	Distribution for sensitivity analysis	Source
Odds ratios				
Odds ratio of PNC for those with ID	2.13			[35]
Odds ratio of MMR for those with ID	0.75			[36]
Odds ratio of MMR for those with PNC	0.90			Assumption
Odds ratio of NMR for those with 4 + ANC visits	0.58			[37]
Odds ratio of NMR for those with ID	0.95			[37]
Odds ratio of NMR for those with PNC	0.79			[37]
Service coverage or impact				
4 + ANC visit baseline coverage	0.34			[25]
ANC impact	0.03	0.046	Beta	[25]
ANC quality of care baseline	0.91			[25]
ANC quality of care impact	0.02	0.028	Beta	[25]
ID baseline	0.91			[25]
ID impact	0.03	0.023	Beta	[25]
ID quality of care baseline	0.60			[25]
ID quality of care impact	0.03	0.036	Beta	[25]
PNC baseline coverage	0.39			[25]
PNC impact	0.03	0.046	Beta	[25]
PNC quality of care baseline	0.07			[25]
PNC quality of care impact	0.03	0.043	Beta	[25]
Birth rate and death rate (show data for 2021 but use data from 2017 to 2021)				
Population size in PBF arm (2021)	23,044,454			
Birth rate (2021)	4.20%			[1]
NMR (2021)	6.24%			[1]
MMR (2021)	0.53%			[1]
Costs				
Cost per ANC visit	17.39	13.48	Gamma	[30]
Cost per ID	97.41	70	Gamma	[38]
Cost per PNC visit	11.31	10.89	Gamma	[30]
Treatment cost of delivery complications	305.284	61.057	Gamma	[38, 39]
Other parameters				
Age at death Pregnant women	27			[40]
Life expectancy at age of death for pregnant women	47.65			[41]
Life expectancy at birth for newborns	62.35			[41]
Discount rate	3%			[33]

ID: Institutional delivery; PNC: Postnatal care; ANC: Antenatal care; MMR: maternal mortality rate; NMR: neonatal mortality rate; PBF: Performance-based financing.

### Sensitivity analysis

A probabilistic sensitivity analysis was conducted for two major parameter categories: (1) costs of health services (e.g., 4 times ANC visits, ID, PNC, and delivery complications); and (2) the PBF’s impact on health service coverage. For parameters lacking reported standard deviations or 95% confidence intervals (CI), we assumed a standard deviation equal to 20% of the mean. We applied a beta distribution for cost parameters and a gamma distribution for the PBF's impact and odds ratios. Using Monte Carlo simulation with 10,000 random draws, we estimated the mean and 95% CI of the ICER.

To incorporate quality of care into the CEA, we created a quality-adjusted coverage indicator for the PBF arm by multiplying service coverage by (1 + the percentage quality improvement) [18]. The DFF arm's quality of care was treated as the baseline (1). We then substituted the standard coverage values for ID, ANC, and PNC with the quality-adjusted coverage values in the decision model, recalculating an ICER that reflected PBF's combined impact on both service quality and coverage. For the quality-adjusted analysis, we calculated the quality-adjusted coverage as the product of coverage and quality improvement percentage, applying this adjustment only to the PBF arm while maintaining the DFF arm's quality of care at 1.

One-way sensitivity analysis was conducted for the impact of PBF on the coverage of three services and the cost of the three services, as well as the treatment cost of complications. We also performed a scenario analysis by creating three scenarios with conservative improvement (0.01), moderate improvement (0.03), and optimistic improvement (0.05) in the coverage of the three services.

Despite many debates [42], we considered the intervention highly cost-effective if the ICER was less than one time per capita GDP of DRC (US$577) and cost-effective if the ICER was less than three times per capita GDP of DRC (US$1,732) using the WHO-CHOICE recommendations [1, 43]. Recent studies remain using one time and three times GDP per capita as the cost-effectiveness threshold [44–46]. Additionally, in resource-constrained settings, such as the DRC with an extremely low GDP per capita, it may be more appropriate to use a generous threshold if a GDP-based threshold is used [47]. A discount rate of 3% was applied to both future costs and benefits in the analysis. All the analysis, including decision tree modelling and sensitivity analysis, was conducted using R 4.3 (The R Foundation, Vienna, Austria).

## Results

### Cost estimation

A total of US$205.9 million (2021 dollar) was spent in the PBF arm over the five-year period (2017–2021), with 70.60% allocated as incentive payments to health facilities and 19.41% as financial transfers to provincial purchasing agencies for contracting PBF facilities and managing the PBF program. The PBF arm covered 100.44 million population-years, resulting in a cost of US$2.05 per person per year.

**Fig. 2:**
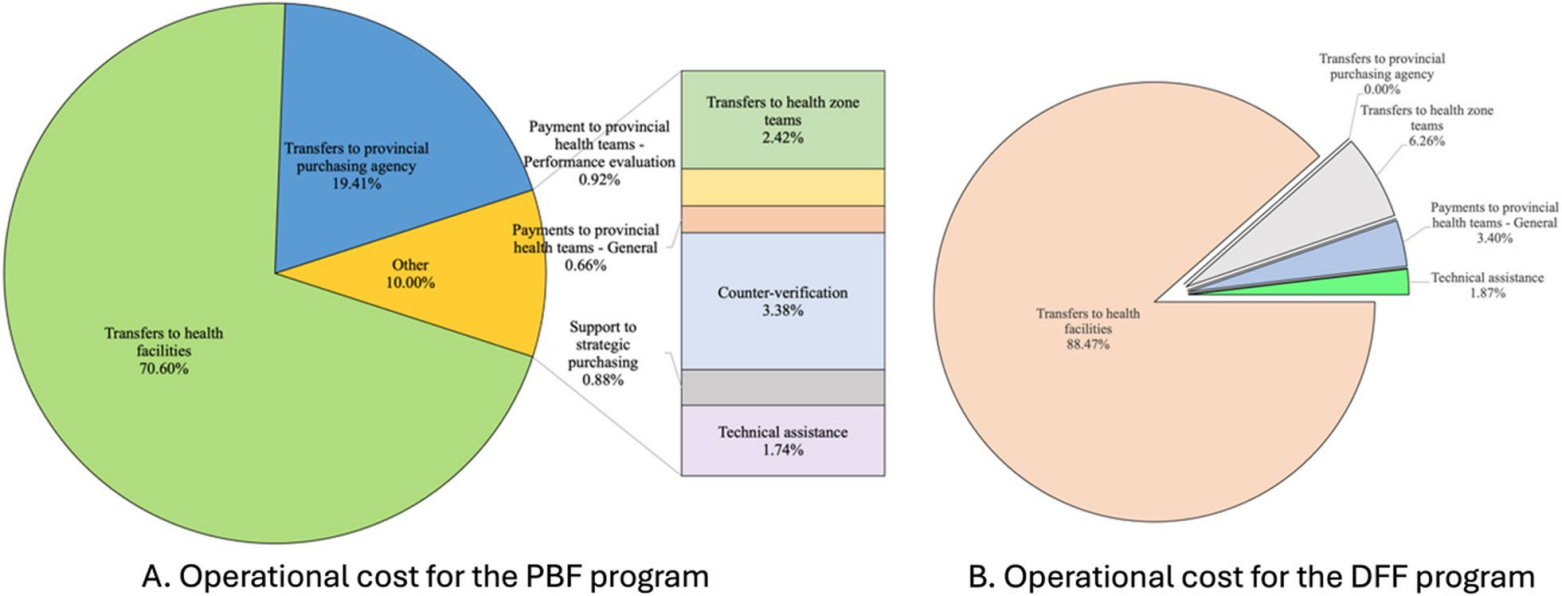
Operational cost distribution for the PBF and DFF arms

In the DFF arm, a total of US$52.2 million was spent in 2021 dollar, with 88.47% used for payment transfer to health facilities (Fig. 2). The population-years covered in the DFF arm were estimated at 30.60 million, yielding a cost of US$1.71 per person per year. Appendix Table 1 presents detailed annual costs, total costs, and per capita costs.

Using the PBF arm's population-years as the standardized measure, the program cost difference was estimated at US$34.53 million, with higher costs in the PBF arm. When accounting for cost differences in service delivery (ANC, PNC, and ID) between arms, the overall cost difference was US$45.34 million (95% CI: US$26.14 million–US$74.81 million), again with higher costs in the PBF arm.

### Effectiveness estimation

Using the decision tree model, increased coverage of ANC, PNC, and ID resulted in lives saved for both pregnant women and newborns. Table 3 presents the annual number of lives saved for pregnant women and newborns, totaling 1,372 over the five years. Newborn lives saved were approximately seven times greater than those of pregnant women.

**Table 3:** Number of maternal and neonatal deaths in PBF and DFF arms for coverage improvement

Year	Maternal deaths			Neonatal death			Total
	PBF arm	DFF arm	Diff (DFF-PBF)	PBF arm	DFF arm	Diff (DFF -PBF)	
2017	4538	4544	6	55,337	55,384	47	53
2018	4426	4443	17	55,101	55,242	141	158
2019	4592	4622	30	55,939	56,177	238	268
2020	4631	4674	42	54,275	54,599	324	367
2021	5156	5217	61	60,423	60,888	465	526
Total	23,344	23,501	157	281,076	282,291	1215	1372

When converted to quality-adjusted life years (QALYs), these lives saved equated to 33,213 QALYs gained (with 95% CI: -41,080 – 120,197). Including quality of care improvements in the analysis increased this figure to 192,036 QALYs gained (95% CI: -144,645 – 712,343). While the point estimates suggested a positive impact of PBF on population life-years, the 95% CIs encompassing zero indicate the possibility that the PBF program might have an opposite effect.

### Incremental cost-effectiveness ratio estimation and sensitivity analysis

**Fig. 3:**
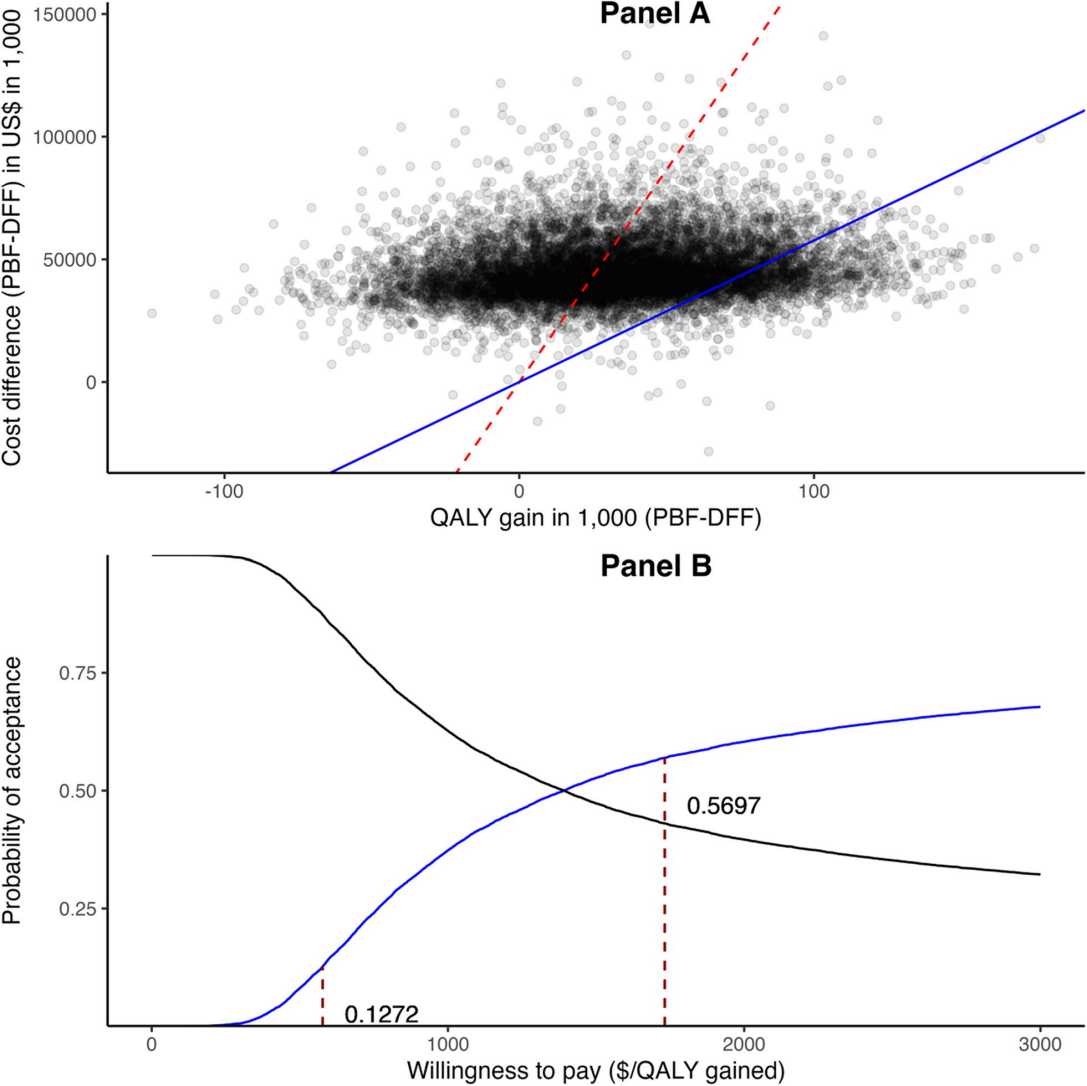
Acceptability curve of the PBF program

The ICER was estimated at $1,374 per QALY (95% CI: -11,881 – 12,566). The acceptability curve without adjusting for the quality of care is presented in Fig. 3. Panel A of Fig. 3 presents the acceptability curve without quality-of-care adjustment. Panel A of Fig. 3 displays a scatter plot of the joint density of incremental costs and incremental effectiveness comparing the PBF and DFF programs. Most data points fell below the dashed line (slope = 1,721, representing three times the per capita GDP threshold), while only a small proportion fell below the solid line (slope = 577, representing one times the per capita GDP threshold).

Panel B further quantifies that the PBF program had a 57.0% probability of being cost-effective using three times per capita GDP (US$1,732) and a 12.7% probability of being highly cost-effective using one time per capita GDP threshold (US$577). When the improvement of quality of care was included in the analysis, the PBF was more cost-effective, with an ICER of US$237.7 per QALY.

**Fig. 4:**
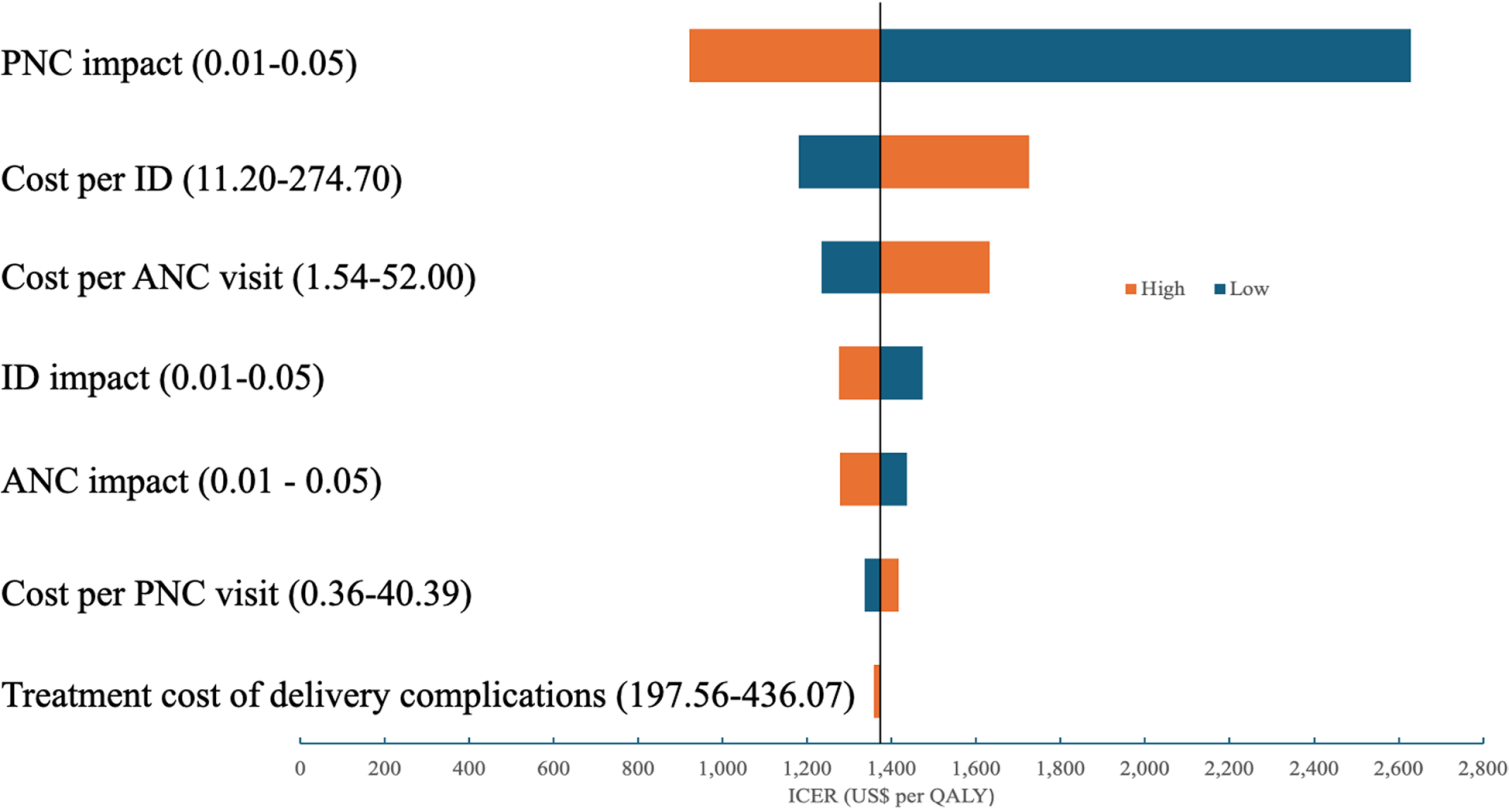
One-way sensitivity analysis

Figure 4 presents the results of the one-way sensitivity analysis. The cost-effectiveness of the PBF program was most sensitive to its impact on PNC coverage, followed by ID costs and ANC visit costs. The treatment cost of delivery complications had minimal impact on the program's cost-effectiveness. In the conservative, moderate, and optimistic scenarios, the ICER was US$3,357, US$1,374, and US$959 per QALY, respectively.

## Discussion

This is the first cost-effective study of the PBF program in DRC using a decision tree model. The model incorporated both empirical findings from the impact evaluation and epidemiological evidence from the literature. We found that the PBF program improves the coverage of MCH services, and it is cost-effective compared to the DFF arm. Without the quality adjustment, the ICER of the PBF program reached US$ 1,374 per QALY. After adjusting for quality, the ICER of the PBF becomes smaller, representing a cost-effective intervention if we use 3 times GDP per capita in 2021 (US$1,732) as the cost-effectiveness cutoff.

The PBF program in the DRC has shown positive impacts on expanding maternal and neonatal health service coverage [25, 26], consistent with findings from other studies [17, 24, 48, 49]. By incentivizing health facilities and workers to improve both service quantity and quality, PBF contributes to increased MCH service coverage. Our analysis revealed annual increases of 1.7% for ANC, 0.6% for ID, and 1.5% for PNC in the PBF group compared to DFF between 2017 and 2021. While these improvements appear modest relative to PBF programs in Nigeria, Zambia, and Zimbabwe [18, 22, 23], this difference likely reflects varying investment levels across programs. For instance, Zambia's PBF program received US$7.91 per capita versus US$2.16 for DFF, while Nigeria's DFF facilities received half of the PBF incentives. In contrast, the DRC's PBF program averaged US$2.05 per capita annually compared to US$1.71 for DFF. These funding disparities explain the smaller observed impact in the DRC, though evidence suggests that enhanced monitoring and supervision can improve service delivery in low-resource settings [18, 23, 25, 26].

This study found that compared to the DFF program, the PBF program in DRC is cost-effective if 3 times per capita GDP is used as the cost-effectiveness threshold. The ICER was estimated at US$1,374 per QALY without quality adjustment, which is similar to those in Zambia (US$1,642 per QALY) and Zimbabwe (US$1,166 per QALY), but higher than those in Nigeria (US$691 per QALY) [18, 22, 23]. The fact that the PBF program is cost-effective shows the value of the conditional payment mechanism, monitoring, evaluation, and supervision on which the PBF program concentrates. However, it should be noted that it is hard to have a pure DFF program as a control group with equal payment disbursement of funding due to various reasons (e.g., reporting is generally mandatory for the PBF program while not necessarily for the DFF program). In this study, the direct payments to health facilities in PBF and DFF arms range from US$1.22–1.76 and US$1.31–1.49 per capita, respectively. The ratio of facility payments per capita to DFF to those to PBF is between 0.81 and 1.17 between 2017 and 2021, with an average ratio of 1.05. Fully controlling for the impact of health resources is not possible. Despite the challenge, the improvement in the coverage of the three essential MCH services can save a significant number of pregnant women (157) and newborns (1215).

It should be acknowledged that the ICER estimated from this study contains a great uncertainty. Partially, it is due to the wide range of 95% CI of the impact of PBF on the coverage and quality of MCH services. To improve ICER towards favorable outcomes for PBF, it is critical to take actions to addressing common implementation barriers in order to sustain the PBF’s improvement towards positive changes, such as strengthening the monitoring of PBF program and facilitating timely disbursement of financial incentives. Additionally, the value of the program could be improved through enhancement of financial efficiency. The verification cost at provincial contracting agencies accounted for about 20% of the total implementation cost in the PBF arm. If the verification cost were reduced by half without compromising the effectiveness of the PBF program, the cost-effectiveness of the PBF program would be improved substantially with an ICER of US$776 per QALY without PBF’s impact on quality.

The PBF program would be more cost-effective if the improvement of quality of care is included in the analysis. Improving the quality of care is another main objective of the PBF program, which is more important when the coverage of essential MCH services is already high. However, the impact of quality of care on ICER should be interpreted with caution. First, the coverage of service may contain the impact of quality of care. In this study, given the high coverage of any ANC visits, we did not use this indicator for the coverage measurement. Instead, we used 4 + ANC visits as the coverage measurement. This indicator often serves as a quality indicator. Second, the impact of PBF on the quality of care for ANC and ID is relatively small, and the large change in ICER after the quality adjustment is mostly driven by the quality improvement in PNC. However, the 45% increase in the quality of PNC may not necessarily be equivalent to the 45% increase in coverage of PNC. Using quality-adjusted coverage simply equates the change in the quality of care to that in coverage of health services and this assumption needs further investigation. Thus, our study focuses on the results without the quality adjustment.

Several limitations of this study should be acknowledged. First, some indicators for MCH services are not included in the modelling. Due to the lack of a clear pathway on how growth monitoring would affect maternal and neonatal mortalities and insignificant improvement from the PBF program, we excluded it from the analysis. We also excluded the use of modern contraceptives from the modelling, due to the lack of information on the specifics. Given the potential positive impact of PBF on the use of contraceptives that would reduce unintended pregnancies and associated deaths and on the growth monitoring, the PBF program would be more cost-effective if it were included [50, 51]. Second, we could not find some country-specific data for the modelling, such as odds ratios and cost information for MCH services. Instead, we used data from the international literature as proxies, and this would result in inaccuracy of ICER estimation. Third, some data were not available, and we had to rely on assumptions. For example, two expenditure items in the direct financing group were either missing or abnormal (disbursement to health facilities in 2019 and transfer to health zone teams in 2018), and we had to extrapolate the expenditure based on the expert’s opinion. Last, but not least, this analysis was conducted from the health system’s perspective, the ICER would be higher if the cost incurred by households were included in the analysis and if the study were conducted from the societal perspective. Despite the potential accuracy concerns, the conclusion that the PBF program is cost-effective remains the same.

## Conclusions

This study utilizes results from the impact evaluation of the PBF program in the DRC to conduct a cost-effectiveness analysis (CEA). The impact evaluation demonstrated PBF's potential to improve coverage of ANC, ID, and PNC services, along with quality of care. Given the program's large population coverage, even modest improvements in these services could make PBF a cost-effective strategy when using three times the 2021 GDP per capita (US$1,732) as the cost-effectiveness threshold. However, the ICER shows substantial variation due to relatively high variability in PBF's impact on these key MCH services. Therefore, maintaining consistent gains in health service coverage from PBF implementation remains crucial.

## Availability of data and materials and code

All the data used in the modelling are available through public sources, and the coding to perform the analysis is available upon request.

## Supplementary Information

Additional file 1. Appendix Table 1.

## Abbreviations

AIDS: Acquired immunodeficiency syndrome
ANC: Antenatal care
CEA: Cost-effectiveness analysis
CI: Confidence interval
DFF: Direct facility financing
DRC: Democratic Republic of the Congo
GDP: Gross domestic product
HIV: Human immunodeficiency virus
ICER: Incremental cost-effectiveness ratio
ID: Institutional delivery
MCH: Maternal and child health
MMR: Maternal mortality rate
NMR: Neonatal mortality rate
PBF: Performance based financing
*PDSS*: *Le Projet de Développement du Système de Santé*
PNC: Post-natal care
QALY: Quality adjusted life year
TB: Tuberculosis
WHO: World Health Organization
