# Supplementary material for: Economic evaluation of financial incentives for maternal and child health in the Democratic Republic of the Congo (DRC): a decision-tree modelling based on a cluster randomized controlled trial
Source: Glob Health Res Policy. 2025 Sep 1;10:41. doi: 10.1186/s41256-025-00435-9 (PMC12400745; doi:10.1186/s41256-025-00435-9)
Supplement: Supplementary file 1 — Additional file 1. Appendix Table 1. [file 41256_2025_435_MOESM1_ESM.docx]

**Appendices**

Appendix Table 1. Cost in PBF and DFF arm in 2021 US$

| Cost items | PBF arm | | | | | |
| --- | --- | --- | --- | --- | --- | --- |
|  | 2017 | 2018 | 2019 | 2020 | 2021 | Total |
| Transfers to health facilities (Million US$) | 22.64 | 26.61 | 29.68 | 35.32 | 31.14 | 145.39 |
| Technical assistance (Million US$) | 0.53 | 0.47 | 0.65 | 0.76 | 0.42 | 3.59 |
| Transfers to health zone teams (Million US$) | 2.11 | 1.24 | 0.49 | 0.54 | 0.61 | 4.98 |
| Payments to provincial health teams - General | 0.19 | 0.33 | 0.30 | 0.30 | 0.24 | 1.36 |
| Counter-verification (Million US$) | 0.00 | 0.45 | 1.05 | 2.87 | 2.59 | 6.96 |
| Support to strategic purchasing (Million US$) | 0.00 | 0.28 | 0.35 | 0.69 | 0.48 | 1.81 |
| Payment to provincial health teams - Performance evaluation (Million US$) | 0.30 | 0.49 | 0.46 | 0.23 | 0.41 | 1.89 |
| Transfers to provincial purchasing agency (Million US$) | 4.63 | 8.06 | 7.86 | 9.40 | 10.01 | 39.96 |
| Total (Million US$) | 30.40 | 37.45 | 40.19 | 49.35 | 45.49 | 205.94 |
| Cost per capita (US$) | 1.64 | 1.98 | 2.02 | 2.47 | 1.97 | 2.05 |
| Cost items | DFF arm | | | | | |
| Transfers to health facilities | 10.02 | 12.63 | 12.65 | 2.90 | 8.01 | 46.21 |
| Technical assistance | 0.14 | 0.13 | 0.18 | 0.21 | 0.11 | 0.98 |
| Transfers to health zone teams | 1.47 | 0.73 | 0.32 | 0.44 | 0.31 | 3.27 |
| Payments to provincial health teams - General | 0.29 | 0.48 | 0.42 | 0.22 | 0.37 | 1.78 |
| Counter-verification | 0.00 | 0.00 | 0.00 | 0.00 | 0.00 | 0.00 |
| Support to strategic purchasing | 0.00 | 0.00 | 0.00 | 0.00 | 0.00 | 0.00 |
| Payment to provincial health teams - Performance evaluation | 0.00 | 0.00 | 0.00 | 0.00 | 0.00 | 0.00 |
| Transfers to provincial purchasing agency | 0.00 | 0.00 | 0.00 | 0.00 | 0.00 | 0.00 |
| Total | 11.93 | 13.84 | 13.39 | 3.56 | 8.69 | 52.23 |
| Cost per capita (US$) | 1.56 | 1.80 | 1.71 | 1.76 | 1.61 | 1.71 |
